# Neural Damage in Experimental *Trypanosoma brucei gambiense* Infection: Hypothalamic Peptidergic Sleep and Wake-Regulatory Neurons

**DOI:** 10.3389/fnana.2018.00013

**Published:** 2018-02-27

**Authors:** Claudia Laperchia, Yuan-Zhong Xu, Dieudonné Mumba Ngoyi, Tiziana Cotrufo, Marina Bentivoglio

**Affiliations:** ^1^Department of Neuroscience Biomedicine and Movement Sciences, University of Verona, Verona, Italy; ^2^Institut National de Recherche Biomédicale (INRB), Kinshasa, Democratic Republic of Congo; ^3^National Institute of Neuroscience (INN), Verona Unit, Verona, Italy

**Keywords:** human African trypanosomiasis, orexin, hypocretin, melanin-concentrating hormone, neuroinflammation, sleep, wake

## Abstract

Neuron populations of the lateral hypothalamus which synthesize the orexin (OX)/hypocretin or melanin-concentrating hormone (MCH) peptides play crucial, reciprocal roles in regulating wake stability and sleep. The disease human African trypanosomiasis (HAT), also called sleeping sickness, caused by extracellular *Trypanosoma brucei* (*T. b*.) parasites, leads to characteristic sleep-wake cycle disruption and narcoleptic-like alterations of the sleep structure. Previous studies have revealed damage of OX and MCH neurons during systemic infection of laboratory rodents with the non-human pathogenic *T. b. brucei* subspecies. No information is available, however, on these peptidergic neurons after systemic infection with *T. b. gambiense*, the etiological agent of 97% of HAT cases. The present study was aimed at the investigation of immunohistochemically characterized OX and MCH neurons after *T. b. gambiense* or *T. b. brucei* infection of a susceptible rodent, the multimammate mouse, *Mastomys* *natalensis*. Cell counts and evaluation of OX fiber density were performed at 4 and 8 weeks post-infection, when parasites had entered the brain parenchyma from the periphery. A significant decrease of OX neurons (about 44% reduction) and MCH neurons (about 54% reduction) was found in the lateral hypothalamus and perifornical area at 8 weeks in *T. b. gambiense-*infected *M. natalensis*. A moderate decrease (21% and 24% reduction, respectively), which did not reach statistical significance, was found after *T. b. brucei* infection. In two key targets of diencephalic orexinergic innervation, the peri-suprachiasmatic nucleus (SCN) region and the thalamic paraventricular nucleus (PVT), densitometric analyses showed a significant progressive decrease in the density of orexinergic fibers in both infection paradigms, and especially during *T. b. gambiense* infection. Altogether the findings provide novel information showing that OX and MCH neurons are highly vulnerable to chronic neuroinflammatory signaling caused by the infection of human-pathogenic African trypanosomes.

## Introduction

Many infections disturb sleep, and somnolence is part of the so-called sickness behavior in response to infections (Poon et al., 2015). The alterations of sleep in human African trypanosomiasis (HAT) are, however, so characteristic and severe that they gave to the disease the alternative name of sleeping sickness (Buguet et al., 2014; Büscher et al., 2017). HAT is caused by the extracellular protozoan parasites *Trypanosoma brucei* (*T. b*.), inoculated through bites of the hematophagous tsetse flies (genus *Glossina*). The disease is still endemic, with a focal distribution mostly in remote rural areas, in the vast sub-Saharan region of the vector’s habitat. After an epidemic in the 1990s, this neglected tropical disease has attracted attention in the public health agenda. Due to sustained control activities, the reported cases have declined in recent years (dropping to less than 3000 in 2015) and the disease is currently targeted for elimination (WHO, 2017). There are, however, concerns on the fight against HAT and worries about disease re-emergence, especially due to inaccurate case reporting and to silent carriers (Welburn et al., 2016; Büscher et al., 2017).

The vast majority of HAT cases (97%; WHO, 2017) are due to the infection of *T. b. gambiense*, for which humans provide the reservoir, causing a chronic progressive form of the disease with a course from 1 to 3 years. *T. b. gambiense* HAT is endemic in western and central Africa, and especially in the Democratic Republic of Congo (DRC) where over 70% of cases in the last 10 years have been reported (WHO, 2017). A more acute form of HAT, lasting 6–8 months, is endemic in eastern and southern Africa and is caused by *T. b. rhodesiense*, representing a zoonosis with wildlife and livestock reservoirs and therefore difficult to control. Most experimental studies on nervous system infection caused by African trypanosomes have been performed using a third parasite subspecies, *T. b. brucei*, which is infectious to animals but not to humans.

During the progression of *T. b*. infection, the parasites, which initially invade peripheral organs through the blood and lymph, enter the central nervous system parenchyma. HAT is considered fatal if untreated (WHO, 2017) and the therapy of the meningoencephalitic stage is very toxic. Ongoing clinical trials based on oral therapy with fexinidazole are opening new perspectives for the cure of HAT (Mesu et al., 2018).

The disease causes severe chronic neuroinflammation, with high levels of inflammatory mediators (Kennedy, 2013; Büscher et al., 2017). Early autoptic studies of HAT victims have shown glial activation with myelin pallor but no features of neurodegeneration (Lejon et al., 2013). The clinical picture, initially nonspecific, leads to a constellation of neurological and psychiatric alterations during disease progression (Kennedy, 2013; Lejon et al., 2013; Buguet et al., 2014; Büscher et al., 2017). Since historical descriptions, striking clinical features of HAT in endemic regions are represented by diurnal somnolence and nocturnal insomnia, as well as episodes of irresistible sleep during wakefulness (sleep attacks; Lhermitte, 1910; Blum et al., 2006; Buguet et al., 2014). Polysomnographic recordings of the sleep-wake cycle in HAT patients have shown that the disease does not cause hypersomnia but rather a circadian disruption of sleep-wake alternation, as well as alterations of the sleep structure (Buguet et al., 2001, 2005, 2014).

Sleep-wake cycle changes during African trypanosomiasis implicate the master circadian pacemaker, the hypothalamic suprachiasmatic nucleus (SCN), which drives endogenous biological rhythms in the mammalian brain (van Esseveldt et al., 2000; Golombek and Rosenstein, 2010; Moore, 2013). Findings on neuronal cell loss in the SCN of an animal model of *T. b. gambiense* infection provided by the multimammate mouse, *Mastomys natalensis* (Mehlitz, 1975; Büscher et al., 2005) are presented in a companion article (Tesoriero et al., 2018). On the other hand, alterations of sleep architecture in African trypanosomiasis implicate damage to structures of the distributed neural network which regulates sleep and wakefulness (Saper et al., 2010; Scammell et al., 2017). In particular, the observation of sudden sleep episodes during wakefulness, as well as sleep fragmentation and disruption of the normal sleep sequence which also occur in *T. b. brucei*-infected rats (Darsaud et al., 2003; Seke Etet et al., 2012; Laperchia et al., 2016, 2017) recall the chronic sleep disorder narcolepsy (Sateia, 2014; Scammell, 2015), whose pathogenesis is due to impaired orexinergic signaling (Liblau et al., 2015). Such alterations point, therefore, to dysfunction of neurons which contain the orexin (OX)/hypocretin peptides, located in the posterior lateral hypothalamus. These neurons play a key role in wakefulness stability and sleep-wake transitions (de Lecea and Huerta, 2014), besides their implication in energy homeostasis and other physiological functions underlying motivated behaviors (Sakurai, 2014; Li et al., 2016).

Orexinergic neurons are intermingled with those expressing another peptide, melanin-concentrating hormone (MCH), which are sleep-promoting and also regulate food intake and other functions implicated in the motivational aspects of behavior (Torterolo et al., 2015; Ferreira et al., 2017). Structural and functional alterations of these two major hypothalamic neuronal populations has been previously reported in *T. b. brucei*-infected laboratory rats and mice (Palomba et al., 2015), but no information on these cell groups during *T. b. gambiense* infection is available. To fill this gap of knowledge, the present study was aimed at the histopathological investigation of OX and MCH cell bodies and OX fibers in the animal model of *T. b. gambiense* infection provided by *M. natalensis*. This rodent is sensitive to *T. b. gambiense* infection (Mehlitz, 1978), at variance with laboratory rodents which are not susceptible to most isolates of this human-pathogenic parasite subspecies (Giroud et al., 2009).

OX neurons give origin to extensive projections (Peyron et al., 1998). In particular, in laboratory rodents orexinergic fibers innervate the area surrounding the SCN (Peyron et al., 1998; Marston et al., 2008), and show a discrete distribution in the thalamus along the midline with dense innervation of the thalamic paraventricular nucleus (PVT; Peyron et al., 1998; McGranaghan and Piggins, 2001; Mintz et al., 2001; Kirouac et al., 2005) documented also in African rodents (Bhagwandin et al., 2011a). The density of OX innervation was here evaluated in these two diencephalic targets of orexinergic projections, the peri-SCN region and PVT, given their high functional relevance for interactions between circadian and vigilance state regulation (Colavito et al., 2015).

## Materials and Methods

### Animals and Infection

Experimental procedures were performed under approval of the ethical committee of the Ministry of Health of DRC, adhering to the European Communities Council (86/609/EEC) directives and the ARRIVE (“Animal Research Reporting of *in vivo* Experiments”) guidelines. All efforts were made to minimize animal number and suffering. The present investigation was based on the same brains of the animals used for the study of the SCN presented in a companion article (Tesoriero et al., 2018).

In brief, adult *M. natalensis* of both sexes (a total number of 45 animals, 30 destined to the two infection paradigms and 15 controls) were obtained from the breeding colony at the Institut National de Recherche Biomedicale (INRB, Kinshasa, DRC).

The rodent *M. natalensis* is very widespread in sub-Saharan Africa, and holds a taxonomic position between the mouse (house mouse) and the rat (ship, roof rat; Coetzee, 1975). The definition of multimammate derives from a uniquely large number of mammae in the female (Isaäcson, 1975). *M. natalensis* (also called *Praomys natalensis*) is nocturnal, with the peak of activity in the first 3 h of the period of darkness (Coetzee, 1975). The animal is omnivorous, uses preferentially burrows of other rodents for nesting and adapts easily to different environments (Isaäcson, 1975), representing the major rodent pest in sub-Saharan Africa (e.g., Mulungu et al., 2013).

The animals were maintained under a 12 h:12 h light/dark cycle, with free access to food and water. One group of animals was infected with *T. b. gambiense* (MHOM/INRB/2006/11A, originally isolated from a patient in DRC in 2006), and another group with *T. b*. *brucei* (AnTat 1.1E). The parasites derived from the cryostabilates collected at INRB, and each experimental group of infected *M. natalensis* included matched uninfected controls. The infection was done with intraperitoneal injection of 0.25 ml per animal of a solution (0.1 M phosphate buffer, pH 7.4, supplemented with glucose) containing 10^6.9^–10^7.2^ trypanosomes/ml. Parasitaemia was verified weekly from the tip of the tail vein. At 4 and 8 weeks post-infection, the animals (*n* = 3 or 4 infected animals per time point and control animals per experimental group) were sacrificed during daytime, under anesthesia, by transcardial perfusion with ice-cold 0.9% saline followed by formaldehyde solution (obtained dissolving paraformaldehyde in 0.1 M phosphate buffer, pH 7.2, at a 4% concentration). Previous monitoring at INRB of the natural course of the infections in *M. natalensis* indicated that at 8 weeks *T. b. brucei* infection is in a very advanced phase (and this was therefore selected as end-point of the study), whereas *T. b. gambiense* infection can last several weeks.

After perfusion, the brains were removed from the skull, posfixed for a few hours, and then stored until processing at 4°C in 0.01 M phosphate buffered-saline, pH 7.4 (PBS), containing 0.1% sodium azide.

### Tissue Processing and Immunohistochemistry

Following cryoprotection in 30% sucrose in PBS, the brains were cut on the coronal plane with a freezing microtome into 30 μm-thick sections, which were collected in six series. One series of sections was stained with cresyl violet for cytoarchitectonic control.

Two series of sections through the hypothalamus were processed free-floating for OX immunohistochemistry. The peptides OX-A and OX-B, also called hypocretin-1 and 2, cleaved from the common precursor prepro-OX, are largely co-localized in the same neurons (Nixon and Smale, 2007), and OX-A is more stable than OX-B. OX-A immunophenotyping was therefore used to investigate orexinergic neurons in the present study. The sections were first soaked in 1% H_2_O_2_ in PBS for 20 min to inactivate endogenous peroxidase activity, and then pre-incubated in a solution of 5% bovine serum albumin (BSA) and 0.3% Triton X-100 in PBS for 1 h at room temperature. Subsequently, the sections were incubated overnight at room temperature in polyclonal goat anti-OX-A antibody (1:500; Santa Cruz, CA, USA) diluted in PBS containing 1% BSA, 0.2% Triton X-100. Following thorough washes, the sections were incubated for 2 h in biotinylated horse anti-goat IgGs (1:200, Vector, Burlingame, CA, USA), and finally reacted with avidin-peroxidase complex (1:100, Vector, Burlingame, CA, USA) for 1 h and with 3,3′-diaminobenzidine (DAB) as chromogen.

The second series was processed further for MCH immunohistochemistry based on a two-color protocol (Peng et al., 1995). These sections were incubated overnight at room temperature in polyclonal rabbit anti-MCH antibody (1:1000; Phoenix Pharmaceuticals, Burlingame, CA, USA). After repeated washing, the sections were incubated for 2 h in biotinylated goat anti-rabbit IgGs (1:200, Vector) and then reacted with avidin-peroxidase complex (1:100; Vector) for 1 h. In the last step of the procedure, the sections were reacted in a freshly prepared and filtered solution containing 0.05% α-naphthol, 0.1% ammonium carbonate, and 0.003% H_2_O_2_ in PBS. The dark blue reaction product was turned into pink by an additional incubation in 0.1% phosphate-buffered pyronin B. This procedure results in the simultaneous visualization in bright-field microscopy of brown DAB reaction products of the first incubation and pink reaction products of the second run (Peng et al., 1995).

After immunohistochemical processing, all sections were thoroughly washed in PBS, mounted on gelatin-coated slides, air-dried, dehydrated, cleared and coverslipped with Entellan. Specific immunostaining was absent in control sections in which the primary antibodies were omitted.

The presence of parasites in the brain parenchyma was investigated in additional sections through the telencephalon and diencephalon, which were processed for double immunofluorescence. These sections were incubated with a mixture of primary antibodies: rabbit polyclonal antibodies which recognize the anti-variant surface glycoprotein of the AnTat 1:1E stabilate (1:200; kindly provided by Philippe Büscher, Institute of Tropical Medicine, Antwerp, Belgium) to visualize the parasites, and goat polyclonal anti-glucose transporter-1 antibodies (1:100, Santa Cruz Biotechnology) to visualize blood vessel endothelia (Pardridge et al., 1990). The sections were rinsed in PBS and incubated in a solution of species-specific secondary antibodies conjugated with Cy2 or Cy3 (1:100; Jackson ImmunoResearch, Suffolk, UK), rinsed in PBS, mounted on slides using a fluorescence-compatible medium (Dako, Hamburg, Germany) and stored at 4°C.

### Data Analysis and Statistics

The sections processed for immunofluorescence for parasite detection were observed with a confocal laser scanning microscope (Zeiss LSM 510 Carl Zeiss, Jena, Germany), equipped with an argon laser emitting at 488 nm (Cy2) and a helium/neon laser emitting at 543 nm (Cy3). The other sections were investigated in bright-field microscopy and quantitative analyses were pursued in three animals per group, blindly of the animal’s experimental group assignment.

The series of sections processed for OX-A single immunohistochemistry was used for OX cell counts, and that processed for double immunohistochemistry for MCH cell counts. The number of OX and MCH cells was determined stereologically using the optical fractionator method throughout the rostrocaudal extent of the perifornical area and lateral hypothalamus. Cells were visualized using an Olympus BX51 microscope (20× objective) with a motorized stage connected to a digital camera (JVC CCD KY-F58) and equipped with the image analysis digital system Stereo-Investigator software (MicroBrightfield Corp.). For a systematic random sampling, a grid centered on the fornix and adjacent medially to the wall of the third ventricle was used. The grid was divided into nine counting frames, allowing also partial cell counts in the medial, middle and lateral portions of the lateral hypothalamus. Only cells within the frame or touching one of the frame borders were counted.

For densitometric evaluation, three regularly spaced sections were sampled through the middle portion of the SCN and PVT, respectively, from each animal. In each section, under calibrated constant light parameters, four images from the PVT and two images from the peri-SCN region (within an area of 300 μm in length lateral to the SCN) were randomly sampled on each side using a 40× objective (NA 0.75; yielding a frame of 0.0256 mm^2^). OX immunoreactivity signal was then measured by defining the zero value of optical density (OD) as background, measured in a portion of the section tissue devoid of specific immunostaining. For each region of interest (ROI), the values from each section were averaged. A grand mean for each ROI was then computed from the mean value derived from each section.

Data are reported as mean ± standard error of the mean (SEM). Kruskal-Wallis analysis of variance followed by the Dunn’s test for pairwise comparison was used for the statistical evaluation of cell counts. One-way analysis of variance followed by the LSD *post hoc* test was used for OD values of immunoreactivity of OX fibers in the peri-SCN region and PVT. Significance threshold was set at *P* < 0.05.

## Results

### Trypanosomes in the Brain Parenchyma

**Figure 1:**
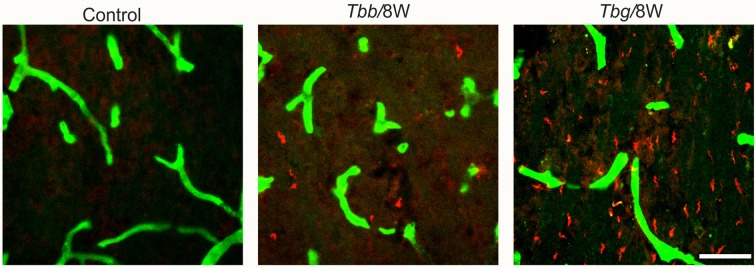
Confocal microscopy images showing parasites (in red) in the brain parenchyma at 8 weeks (W) after *Trypanosoma brucei brucei* (*Tbb*) or *Trypanosoma brucei gambiense* (*Tbg*) systemic infection. Blood vessel walls are visualized by anti-glucose transporter-1 immunolabeling (in green). Scale bar: 40 μm.

Double immunofluorescence showed in *M. natalensis* the occurrence of *T. b. gambiense* and *T. b. brucei* outside blood vessels within the brain parenchyma, in variable amounts, at both 4 weeks and 8 weeks (Figure 1) after infection, as also presented in the companion article (Tesoriero et al., 2018). This indicated that in both paradigms the infection was in the encephalitic stage at the sampled time points.

### OX and MCH Cell Bodies

**Figure 2:**
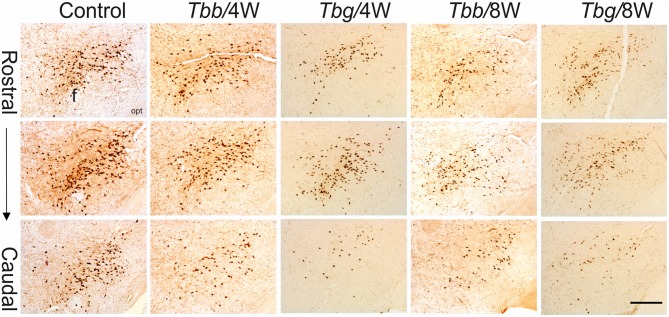
Low power view of orexin (OX)-A-immunoreactive neurons in the perifornical area and lateral hypothalamus of *Mastomys natalensis* at 4 weeks (W) and 8 W after infection with *Trypanosoma brucei brucei* (*Tbb*) or *Trypanosoma brucei gambiense* (*Tbg*) and in uninfected animals. Note the rarefaction of immunostained cell bodies and the reduction of neuropil immunolabeling in the infected animals. Abbreviations: f, fornix; opt, optic tract. Scale bar: 250 μm.

**Figure 3:**
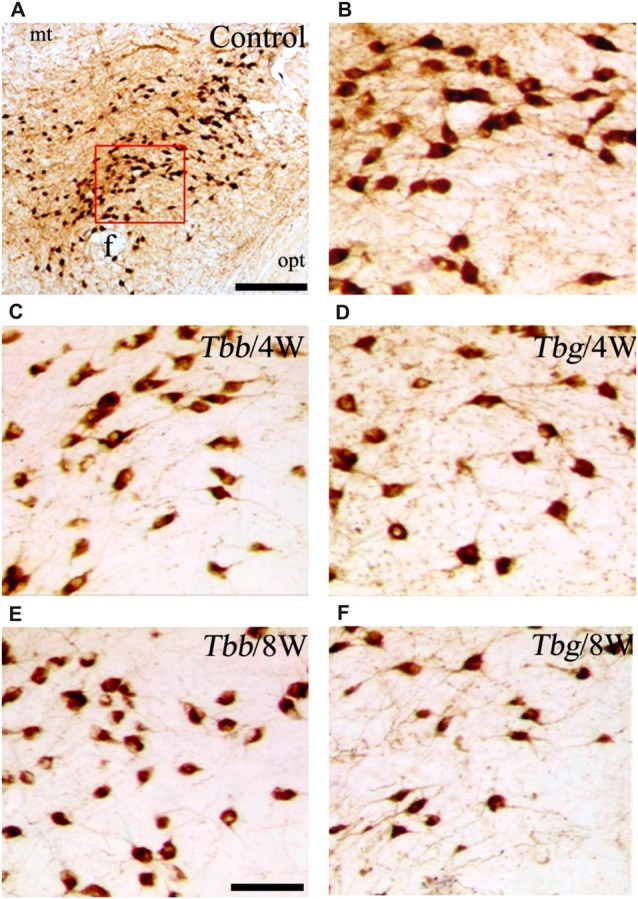
**(A,B)** Images of orexin-A-immunoreactive neurons in uninfected *Mastomys natalensis* (**B** represents at higher power the area boxed in **A**), and at 4 weeks (W) and 8 W after infection with *Trypanosoma brucei brucei* (*Tbb*) or *Trypanosoma brucei gambiense* (*Tbg*) **(C–F)**. Abbreviations: f, fornix; opt, optic tract. Scale bars: 250 μm in **(A)**, 150 μm in **(E)** (applies also to **B–D,F**).

**Figure 4:**
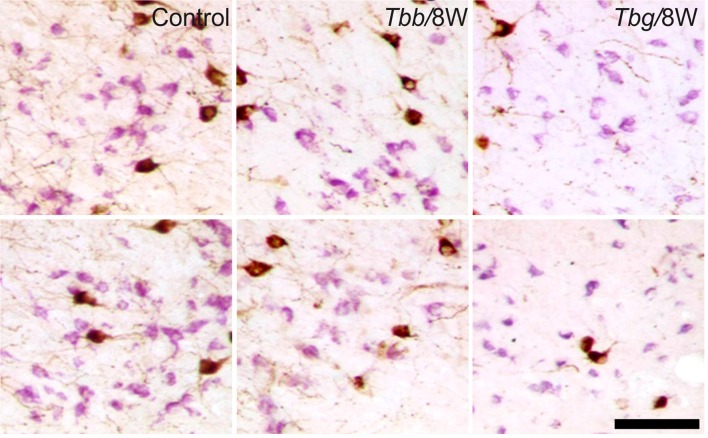
Images of OX-A-immunoreactive neurons (brown) and melanin-concentrating hormone (MCH)-immunoreactive neurons (purple) through the middle third (upper row) and posterior third (lower row) of the lateral hypothalamus of uninfected *Mastomys natalensis* and at 8 weeks (W) after infection with *Trypanosoma brucei brucei* (*Tbb*) or *Trypanosoma brucei gambiense* (*Tbg*). Note the marked shrinkage of many MCH-immunostained neurons. Scale bar: 150 μm.

In *M. natalensis*, OX-immunolabeled neurons showed a distribution similar to that described in laboratory rodents (Peyron et al., 1998; Mintz et al., 2001) and in a variety of African rodents (Bhagwandin et al., 2011a,b; Sweigers et al., 2017). The largest population was concentrated in the perifornical area, from which it extended to the lateral hypothalamus (Figure 2). MCH neurons also showed in the hypothalamus the distribution described in rodents, very similar, in particular, to that reported in the rat (Bittencourt, 2011). Thus, MCH neurons were distributed rostrally in the incerto-hypothalamic area, between the fornix and the internal capsule at the level of the tuberal lateral hypothalamus. Proceeding posteriorly, MCH neurons were densely aggregated around the fornix, and with sparser distribution medially to the internal capsule, as well as dorsally in the zona incerta, and with a medial, periventricular location. MCH neurons were intermingled with OX neurons in the perifornical area and lateral hypothalamus, and also surrounded OX neurons dorsally and ventrally. OX and MCH somata were morphologically similar, with a multipolar or fusiform shape (Figures 3A,B, 4), giving origin to varicose fibers.

A decrease in the density of OX cell bodies and neuropil immunostaining after African trypanosome infection at both the sampled time points was evident even at low power observation along the rostrocaudal and mediolateral axes (Figure 2). At higher power, the intensity of the soma immunostaining appeared relatively preserved in the infected animals, but with less extensive dendritic filling than in matched controls, and some cell bodies appeared shrunken (Figures 3C–F). Also the density of MCH neurons appeared decreased in both paradigms of infection, with shrinkage of many cell bodies (Figure 4).

**Figure 5:**
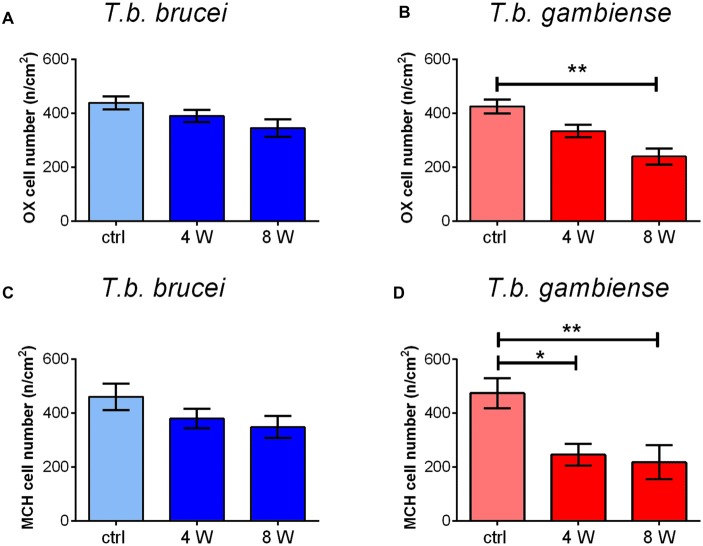
The bar graphs illustrate the stereological counts of neurons containing OX-A **(A,B)** or MCH **(C,D)** in control (ctrl) *Mastomys natalensis* and at 4 weeks (W) and 8 W after infection with *Trypanosoma brucei* (*T. b.*) *brucei* **(A,C)** or *T. b. gambiense* **(B,D)**. *P < 0.05; **P < 0.001.

Cell counts documented a progressive decrease of both peptidergic cell populations, which was more marked after *T. b. gambiense* infection than in the *T. b. brucei*-infected animals (Figure 5). In particular, after *T. b. brucei* infection the reduction of OX-immunostained cells with respect to controls was 11.12 ± 6.8% at 4 weeks and 21.34 ± 9.8% at 8 weeks, and that of the MCH-immunostained cells, evaluated in the lateral hypothalamus and perifornical area, was 17.53 ± 10.6% at 4 weeks and 24.45 ± 2.2% at 8 weeks. These decreases did not reach, however, statistical significance. After *T. b. gambiense* infection, 21.58 ± 7.6% reduction of OX-immunostained cells was documented at 4 weeks, and 43.78 ± 7.4% reduction at 8 weeks, when the cell number decrease vs. matched controls was significant. Even more marked was the decrease of MCH neurons (48.15 ± 4% reduction at 4 weeks and 54.15 ± 6.3% at 8 weeks), which was significant at both survival times.

Partial cell counts showed that loss of immunostained cell bodies occurred throughout the extent of their distribution in the lateral hypothalamus and perifornical area.

### OX Fibers

**Figure 6:**
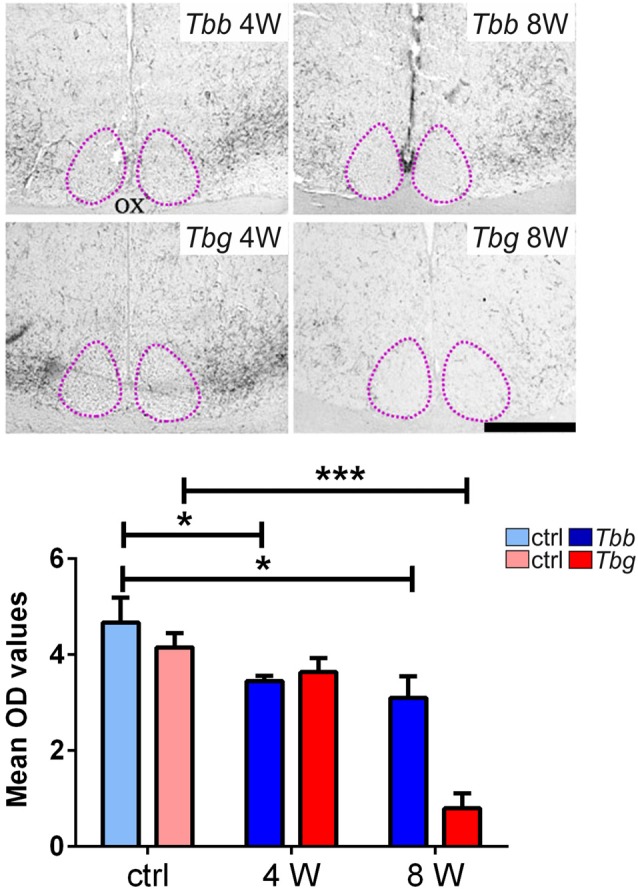
Top: images of the ventral part of the anterior hypothalamus showing OX-A immunoreactive fibers in the region lateral to the suprachiasmatic nucleus (SCN, delimited by adotted line), in *Mastomys natalensis* at 4 weeks (W) and 8 W after infection with *Trypanosoma brucei brucei* (*Tbb*) or *Trypanosoma brucei gambiense* (*Tbg*). Note that the decrease in the immunoreactive fiber density, especially at 8 weeks after *T. b. gambiense* infection. Scale bar: 250 μm. Bottom: bar graphs of the quantitative densitometric evaluation of OX-A immunosignal in the peri-SCN region in control (ctrl) and infected animals. *P < 0.05; ***P < 0.001. Abbreviations:OD, optical density; OX, optic chiasm.

**Figure 7:**
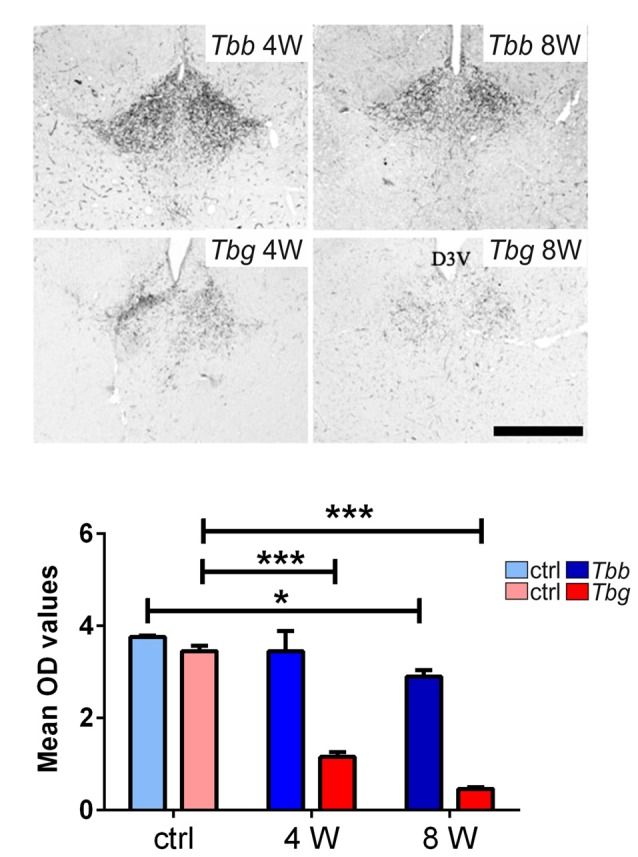
Top: images of the paraventricular thalamic nucleus (PVT) of *Mastomys natalensis* showing OX-A-immunoreactive fibers at 4 weeks (W) and 8 W after infection with *Trypanosoma brucei brucei* (*Tbb*) or *Trypanosoma brucei gambiense* (*Tbg*). Note the marked decrease of PVT orexinergic innervation, especially after *T. b. gambiense* infection (see also Figure 6). Scale bar: 250 μm. Bottom: bar graphs of the quantitative densitometric evaluation (OD, optical density) of OX-A immunosignal in PVT of control (ctrl) and infected animals. *P < 0.05; ***P < 0.001.

**Figure 8:**
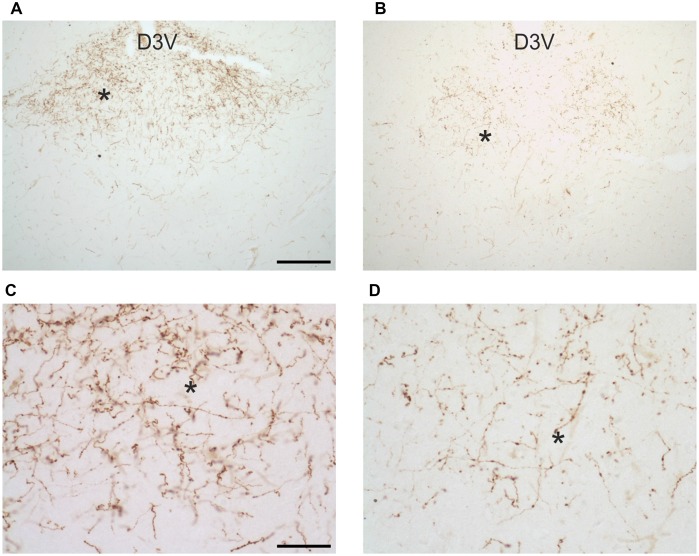
Images of orexin-A-immunoreactive preterminal and fibers in the paraventricular thalamic nucleus of uninfected *Mastomys natalensis* **(A,C)** and at 8 weeks after infection with *Trypanosoma brucei gambiense* **(B,D)**. **(C,D)** represent at higher magnification fields of **(A,B)**, respectively, as indicated by the asterisks for spatial landmark. Note in **(C)** the abundance of varicose fibers in the control animals and note in **(D)** their marked decrease and fragmentation in the infected animal. Abbreviation: D3V, dorsal third ventricle. Abbreviation: D3V, dorsal third ventricle. Scale bars: 200 μm in **(A,B)**, 50 μm in **(C,D)**.

In uninfected *M. natalensis*, orexinergic fibers were found to be densely aggregated laterally to the SCN, as well as along the thalamic midline, innervating PVT throughout its extent. A significant density decrease was found in the orexinergic innervation of the peri-SCN region at 4 and 8 weeks after *T. b. brucei* infection (Figure 6). In PVT, *T. b. brucei* infection resulted in a reduction of orexinergic fiber density at 4 weeks, which was further reduced, and significant, at 8 weeks (Figure 7). In the peri-SCN region of *T. b. gambiense*-infected animals the decrease in the density of orexinergic fibers was very marked and highly significant at 8 weeks (Figure 6). Extreme reduction of orexinergic innervation was found in PVT at both 4 and 8 weeks after *T. b. gambiense* infection (Figures 7, 8).

## Discussion

The present findings show that *T. b. gambiense* infection leads to progressive quantitative decrease of OX and MCH neurons, and that this is more marked than that caused by *T. b. brucei* infection in the same host species, susceptible to both parasite subspecies. At the endpoint of the study (8 weeks post-infection), the reduction of OX neurons in *M. natalensis* was about 44% and that of MCH neurons accounted for the loss of about 54% of these neurons in the lateral hypothalamus and perifornical area. Of note, considerable damage of MCH neurons was documented in *M. natalensis* also at an earlier phase of the encephalitis (about 48% reduction of MCH neurons and about 21% reduction of OX neurons at 4 weeks post-infection).

Immunophenotyping could not here reveal whether the decrease was due to cell death phenomena or downregulation of peptide expression below the threshold of immunohistochemical visualization. However, in the infected animals immunostaining showed cell damage suggestive of an ongoing degenerative process, previously documented also in *T. b. brucei*-infected laboratory rodents in the absence of overall cell loss in the hypothalamus (Palomba et al., 2015).

The present analyses in infected *M. natalensis* also demonstrated significantly impoverished orexinergic innervation, which was especially marked after *T. b. gambiense* infection. This was here shown in the peri-SCN region, which is in turn involved in the circadian control of OX neuron activation (Marston et al., 2008; Belle et al., 2014; Belle and Piggins, 2017) that could be thus impaired during the infection. Decreased orexinergic input was also here documented in PVT, the thalamic midline structure in which OX release exerts powerful functional effects, and which plays a key role in funnelling state-dependent behavior information into the limbic system and prefrontal cortex (Colavito et al., 2015).

Loss of OX neurons (90% or greater) is the neuropathological hallmark of narcolepsy, and in particular of narcolepsy with cataplexy (sudden loss of postural muscle tone, triggered especially by emotion; Peyron et al., 2000; Thannickal et al., 2000). OX neurons degenerate also in narcolepsy without cataplexy, as reported in a *post-mortem* brain examination which showed loss of 33% of OX neurons (Thannickal et al., 2009). Besides narcoleptic-like changes of sleep architecture in HAT, the clinical phenotype of the sleep-wake cycle is, however, very different in this disease and in narcolepsy, since narcolepsy does not include circadian rhythm disturbances (Dantz et al., 1994).

The investigation of OX levels in the cerebrospinal fluid (CSF) of *T. b. gambiense* HAT patients (Dauvilliers et al., 2008) and *T. b. brucei*-infected rats (Palomba et al., 2015) have shown a decrease, which, however, was not significant and exhibited high interindividual variability. The concentration of OX in the CSF of narcoleptics with typical cataplexy is very low, whereas it is normal in most cases of narcolepsy without cataplexy, as well as in other neurodegenerative or neurotraumatic conditions which lead to partial loss of OX neurons (Bourgin et al., 2008). On the other hand, experimental evidence based on lesions of OX neurons in the rat has shown that a very large cell loss is needed to impair OX level in the CSF, which points to a considerable compensatory capacity of surviving OX neurons (Gerashchenko et al., 2003).

Importantly, polysomnographic recording of a limited cohort of *T. b. gambiense* HAT patients has shown that the narcoleptic-like sleep structure alterations can slowly recover after trypanocydal therapy, especially when patients are not severely ill (Buguet et al., 1999). This suggests that functional compensation can be effective when the disease is promptly cured.

The present finding of MCH neuron damage shows that *T. b. gambiense* infection does not affect OX neurons selectively. This also occurs in other conditions. For example, sleep disorders represent prominent non-motor symptoms in Parkinson’s disease, and loss of both OX and MCH neurons, increasing with disease severity, has been reported in *post-mortem* studies of the hypothalamus of victims of this disease (Thannickal et al., 2007). It is relevant to recall, in this respect, that MCH neurons are instead spared in the brain of narcoleptic patients with or without cataplexy (Peyron et al., 2000; Thannickal et al., 2000, 2009). Altogether the findings indicate that *T. b. gambiense* infection disrupts the interplay between OX and MCH neurons, supporting data obtained in laboratory rodents after *T. b. brucei* infection (Palomba et al., 2015). These peptidergic neurons not only play reciprocal roles in the regulation of vigilance states (Konadhode et al., 2015), but are also interconnected. Notably, MCH neurons exert an inhibitory influence on OX neurons, tuning the overall output of these two systems (Rao et al., 2008).

Interestingly, the reduction of OX and MCH neurons here documented in *T. b. gambiense* infection is much more marked than that provoked by local (intra-hypothalamic) infusion of the endotoxin lipopolysaccharide for 1 month (Gerashchenko and Shiromani, 2004). Experimental studies based on *T. b. brucei* infection have indicated an escalating inflammatory response during disease progression (Kristensson et al., 2010) in which the posterior hypothalamus is especially involved (Laperchia et al., 2016). Parasites reside in the median eminence, as in other circumventricular organs, since the first stage of hemolymphatic infection (Kristensson et al., 2010). The posterior hypothalamus is an early site of parasite traversal of blood-brain barrier through gradients of permeability from the median-eminence-arcuate nucleus complex (Laperchia et al., 2016). This hypothalamic complex is known to represent a first order station for peripheral signals, transmitting information to the second order network provided by OX and MCH neurons (Guyon et al., 2009; Rostène et al., 2011). It may therefore not be surprising that these peptidergic cell populations are targeted by chronic inflammatory signaling during African trypanosomiasis.

Several data sets point to vulnerability of OX (Grossberg et al., 2011) and MCH (Sergeyev et al., 2001) neurons to inflammatory molecules. OX neurons are especially sensitive to nitric oxide toxicity (Obukuro et al., 2013). MCH neurons are very sensitive to signaling mediated by the CCL2 chemokine (Le Thuc et al., 2016). The expression of this chemokine in the brain during African trypanosomiasis remains to be explored, but CCL2 is part of the panel of inflammatory mediators increased in the CSF of *T. b. gambiense* HAT patients (Hainard et al., 2009).

In conclusion, the present findings show that sleep-wake-regulatory OX and MCH neurons are vulnerable to the infection caused by a human-pathogenic parasite responsible for the vast majority of HAT cases, and indicate that MCH neurons are especially susceptible to this infection. HAT causes a paradigmatic chronic progressive neuroinflammation, and the present data could therefore have pathogenetic implications for sleep disorders in other chronic neuroinflammatory conditions. Concerning HAT, the data recall the importance of an early diagnosis and therapy to favor compensation and recovery of vulnerable neuronal cell types damaged during the disease.

## Author Contributions

CL and Y-ZX: data analysis and manuscript preparation. Y-ZX: tissue processing. DMN: infection design and animal experiments. TC: manuscript preparation: MB: study design and article writing.

## Conflict of Interest Statement

The authors declare that the research was conducted in the absence of any commercial or financial relationships that could be construed as a potential conflict of interest.
